# Alleviating Head-mounted Weight Burden for Neural Imaging in Freely-behaving Rodents Using a Helium-filled Balloon

**DOI:** 10.21203/rs.3.rs-5247340/v1

**Published:** 2024-11-20

**Authors:** Yuehan Liu, Jing Zhang, Cheng-Yu Li, Haolin Zhang, Xingde Li

**Affiliations:** Johns Hopkins University; Johns Hopkins University; Johns Hopkins University; Johns Hopkins University; Johns Hopkins University

## Abstract

The recently developed miniaturized head-mounted two-photon (2P) imaging devices have served as a valuable tool for neuroscientists, enabling real-time functional neural imaging in freely-behaving animals. Although the current 2P fiberscopes and miniscopes are lightweight, the weight of any potential additional accessories inevitably imposes a burden on the animal. Here, we present a buoyancy levitation method to alleviate head-mounted weight burden on mice. By utilizing the buoyance of a helium-filled balloon to counteract the additional weight of up to 7 g, both the motion behavior and neural activities remain unaffected by the added load. This easy-to-implement method provides a platform for studying neural network function in animals, effectively freeing them from the burden of head-mounted weight.

## 1. Introduction

Two-photon (2P) fluorescence microscopy has served as a valuable tool in neuroscience for investigating dynamic neural networks with high spatiotemporal resolution [1–3]. Along with genetically encoded calcium indicators, particularly the family of GCaMP, benchtop 2P microscopy has been widely used to optically assess neural activities in head-fixed animals [4–7]. While effective, these protocols are suboptimal for studying nonstationary behaviors such as grooming, spatial navigation, social interaction. Additionally, head fixation induces pressure and fatigue in the head and neck, which can lead to aberrant neural feedback, and interfere with the study of neural circuit function.

To address these limitations, miniaturized, head-mountable 2P imaging devices have been developed, allowing the visualization of brain activities and neural network dynamics in freely-behaving animals [8–10]. Over the past two decades, significant efforts have been invested to reduce the size and weight of 2P miniscopes [11–13]. Recently developed ultralight ultracompact 2P fiberscopes (weighing 1 g, with an outer diameter of 2.4–2.8 mm) have enabled neuroimaging in freely-walking/rotating mice [14–18]. However, despite the lightweight design of current 2P fiberscopes and miniscopes, additional optics or mechanical components may need to be attached to the imaging probe for specific imaging control purposes, such as light-targeting devices for optogenetics control [19, 20]. This added weight of the accessories inevitably places a burden on the animal’s head, potentially affecting its behavior.

In this study, we developed and demonstrated a buoyancy levitation method to alleviate the head-mounted weight burden in freely-behaving mice. By introducing a buoyant force to the head-mounted fiberscope using a standard helium balloon (15 L), we demonstrated the weight cancellation of the additional accessories on the mouse’s head, covering a range of 0 – 7 g. Motion behavioral analysis revealed that the mice exhibited similar freedom of walking (in terms of walking distance and turning angles) when the balloon was used to lift the head-mounted weight, compared to when only the bare fiberscope was worn by the mice. Furthermore, the animal’s neural behavior (firing activities) remained consistent with the application of buoyant force, indicating that the weight burden once lifted by the balloon did not interfere with neural activities. We believe this convenient method offers a practical solution to reduce the head-mounted weight on animals and enable neural network function study when the animal is under naturalistic conditions.

## 2. Methods

**Figure 1:**
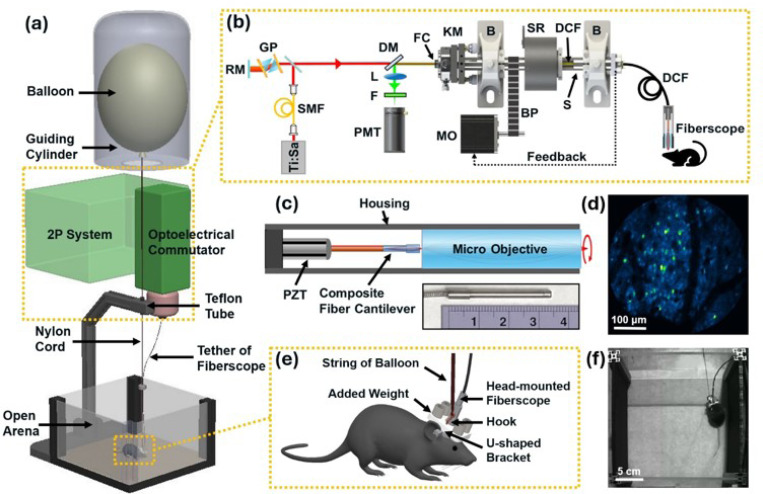
The buoyancy levitation system for 2P neural imaging in freely-behaving mice. (a) Schematic of the buoyancy levitation system. (b) Schematic of optoelectrical commutator along with 2P fiberscope imaging system. SMF: single-mode fiber; RM: roof mirror; GP: GRISM pair for dispersion compensation; DM: dichroic mirror; L: lens; F: optical filter; PMT: photomultiplier tube; FC: fiber collimator; KM: kinematic mount; MO: stepper motor; B: bearing; BP: belt/pulley; SR: slip ring; DCF: double-clad fiber. (c) Schematic of a 2P fiberscope. (d) Representative 2P image of neurons expressing GCaMP6m, obtained from a fiberscope. (e) Zoomed-in diagram of the mouse’s head with a 2P fiberscope and an additional weight. (f) Photograph of a mouse in an open arena with a head-mounted fiberscope, additional weight, and a balloon counteracting the weight.

To reduce the head-mounted weight burden imposed on the freely-behaving mice, we developed a buoyancy levitation system for 2P neural imaging. The schematic of the system is shown in Fig. 1a, where a helium balloon, filled with an 80% helium − 20% air mixture, provides lifting buoyancy to counteract the additional weight placed on the mouse’s head. A guiding cylinder is positioned above to restrict the lateral movement of the balloon, thereby minimizing the air resistance when the animal moves and the associated vertical movement of the balloon. A piece of Teflon tubing is installed at the connection point between the nylon cord and the stand to reduce friction and further ensure the vertical movement of the balloon. Figure 1b illustrates the block diagram of our 2P imaging platform, where the 2P fiberscope is connected to an electrical commutator for rotational tracking and compensation, allowing neuroimaging in freely-walking/rotating mice [16]. Figure 1c shows the schematic of our 2P fiberscope with the photograph of a fully assembled fiberscope. The fiberscope is based on fiber-optic scanning technology [17, 21] and our recent composite fiber cantilever design for obtaining a large field of view (FOV) [14]. Figure 1d provides a representative 2P GCaMP6m neuroimage acquired with the fiberscope from a freely-behaving mouse. Zoomed-in diagram of the mouse’s head is shown in Fig. 1e, where a 3D-printed lightweight U-shaped bracket (0.5 g) is designed for easy mounting of additional weight (e.g., stainless metal rods). The balloon could hook onto this U-shaped bracket to provide buoyancy without interfering the attachment of the fiberscope. Figure 1f shows a mouse in the arena with a head-mounted fiberscope and a balloon counteracting the additional weight.

The buoyancy of the helium balloon F can be calculated using the following equation:

$$F=\Delta \rho •g•V=\left(\rho_{air}-\rho_{balloon}\right)•g•V$$

where $\rho_{air}$ is the density of air, $\rho_{balloon}$ is the density of the gas inside the balloon (80% helium − 20% air mixture), g is the gravitational acceleration, and V is the volume of the balloon.

A standard party balloon can be inflated into an approximately 15L ellipsoid. According to our calculation, the buoyancy it provides can lift around 12 g of weight. The balloon itself weighs 3 g, and with the addition of a nylon cord and a hook, the total weight is approximately 5 g. Therefore, the buoyancy levitation system with a 15L balloon is capable of offsetting an additional weight of up to 7 g. A helium balloon of large size could be used to lift a heavier weight.

**Figure 2:**
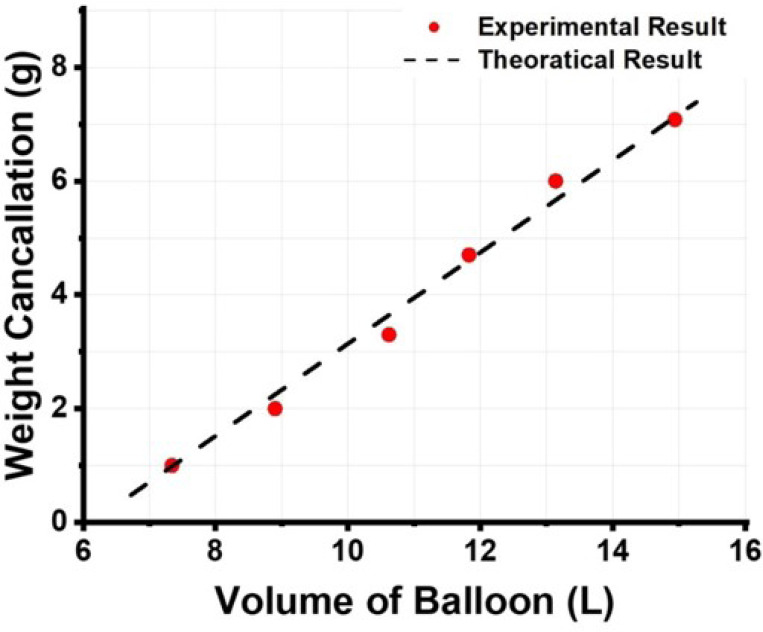
The relationship of weight cancelled by the buoyance verse the volume of the helium balloon. The red dots show the experimental results, and the dashed line illustrates the theoretically calculated values.

Figure 2 plots the relationship between the weight cancelled by the buoyancy and the volume of the balloon, showing both the theoretically calculated values and the experimental measured results. The measurements were performed by placing weights on a precision electronic scale. The weight cancellation was confirmed only when the scale read 0.0 g, with a helium balloon lifting the weight. The volume of the balloon was determined by measuring its diameter and height. The figure illustrates that the actual measurement results closely match the theoretical predictions, with minor deviations likely due to inaccuracies in measuring the balloon’s dimensions.

### Animals

Three Camk2-Cre line mice (Jax, #005359) with GCaMP6m expressed in the somatosensory cortex were used in this work. All experimental studies were in accordance with ARRIVE guidelines. All experimental methods were approved by the Johns Hopkins University Animal Care and Use Committee under animal protocol #MO21M4111. All methods were performed in accordance with the relevant guidelines and regulations.

### Cranial window surgery

At the age of 8-weeks old, the mice were anesthetized by inhalation of 2% isoflurane and O_2_ and then locked to a stereotaxic platform. After removing the scalp and exposing the skull, a 4 mm diameter round craniotomy was drilled over the somatosensory cortex of the mice. AAV/DJ-flex-GCaMP6m virus (Neuroconnectivity Core, Baylor College of Medicine) was injected into the target region (−2.5 mm lateral, −1.2 mm anterior to the bregma, 0.55 mm depth) via a glass microneedle (World Precision Instrument TIP10LT, 1mm O.D., 10 μm tip diameter) attached to a microinjector pump (Nanoject II, Drummond). After the injection, a 100 μm thickness glass coverslip was placed to the exposed brain, and it was sealed to the skull with tissue adhesive (3M Vetbond). A customized titanium head-restraining bar was glued to the head with dental cement for later attaching the endomicroscope. The transgene expression was checked 3–4 weeks post surgeries with a tabletop 2P microscope. Enthanasia (CO_2_) would be performed only if the cranial window showed signs of infection (e.g., cloudy window) or the condition or health of the mouse degraded beyond recovery. Every effort was made to reduce the number of animals used, and to minimize their suffering.

### Freely-behaving mouse neural imaging

After the transgene expression was confirmed, the mice would be used for 2P fiberscopy imaging *in vivo*. To begin the imaging procedure, the mouse was lightly anesthetized (0.5–2% Isoflurane + 1.5L/min oxygen) and restrained by locking its head-restraining bar to a home-made holding platform. Then a 2P fiberscope was mounted on a 3D translational stage and carefully positioned above the cranial window to image the brain. After a suitable FOV was identified, the fiberscope was fixed onto the head-restraining bar via a customized light-weight mini-adapter, the mouse was then released from the holding platform and started moving freely in a home-built imaging platform (10” × 10” open arena). The mouse was allowed sufficient time to recover from anesthesia before freely-behaving neural imaging experiments. One camera (BFLY-PGE-12A2M-CS, FLIR) was set above the imaging platform to obtain the top view of the freely-behaving mouse. The 2P imaging data from the fiberscope and behavior recordings from the camera were collected in synchronization and saved for later analysis. After imaging, the mouse will be anesthetized again, and the fiberscope was detached from the mouse. Afterwards, the mouse will be placed in a housing facility for at least 24 h of rest before the next imaging experiment.

## 3. Results

We tested the performance of the buoyancy levitation system by conducting neural imaging in freely-behaving mice under three conditions: (1) Probe only: Only a 2P fiberscope was mounted on the mouse’s head. (2) Balloon + Weight: In addition to a fiberscope, an additional 6 g additional weight was mounted on the mouse’s head, with a helium balloon providing enough buoyancy to cancel out the added weight. (3) Weight: A fiberscope and a 6 g additional weight were mounted on the mouse’s head, but without any buoyant lift.

During the experiment, each mouse was set free in an open arena for a 2P imaging session of 500 frames (3 frames/s, ∼167 s per session) under each of the three conditions. Between sessions, there was an intersession break of at least 5 minutes to allow the animal sufficient rest. The animal’s motion behavior was recorded using a camera and the walking trajectories were extracted from the videos using DeepLabCut [22], a toolbox for motion tracking. From these trajectories, the total walking distance and turning angle of the animal were calculated. Real-time *in vivo* neural images over the somatosensory cortex (expressing GCaMP6m) of the mouse were acquired using the 2P fiberscope. The neural data was processed using a well-established pipeline CaImAn [23], along with Python-based custom codes. The experiments were performed on three mice. Both their motion behavior and the neural activities were analyzed.

**Figure 3:**
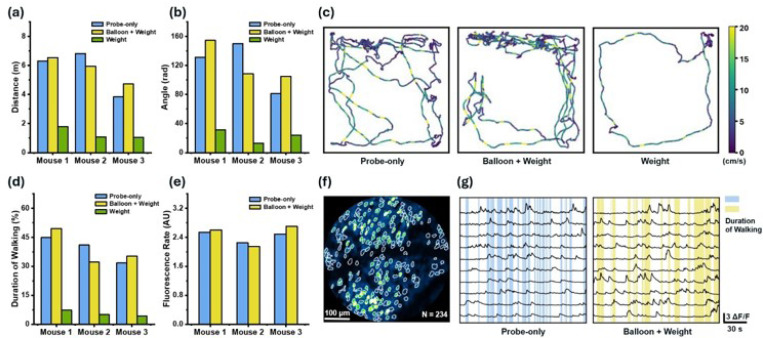
(a) Total walking distance within a session under different conditions (blue: probe-only, yellow: balloon + weight, green: weight) of three mice (Mouse 1, Mouse 2, and Mouse 3). (b) Total turning angle within a session under different conditions for the three mice. (c) Representative movement trajectories of a mouse under different conditions. Color implies walking speed. (d) The percentage of walking time relative to total imaging session under different conditions. (e) Average fluorescence rate of the identified neurons during the walking duration. The fluorescence rate of a neuron is defined as the area under its ΔF/F trace during walking divided by the walking duration. (f) Segmentation mask of 234 neurons identified in the FOV of Mouse 1’s somatosensory cortex. (g) Representative ΔF/F traces of 10 neurons identified in (f). The blue and yellow shading represent the walking duration under “probe-only” and the “balloon + weight” conditions, respectively.

The total walking distance during each session for all three mice under the different conditions is shown in Fig. 3a, and the total turning angle is given in Fig. 3b. The results indicate that with a helium balloon alleviating the additional head-mounted weight burden, all three mice exhibited a similar freedom of movement compared with only wearing a fiberscope. Without the buoyance from the balloon, however, the mice were burdened by the additional weight and walked much shorter distances. Figure 3c shows representative movement trajectories of a mouse, demonstrating that the mice were much more active under the “probe-only” and “balloon + weight” conditions. We also calculated the percentage of walking time relative to the total imaging session duration under the different conditions. The results (Fig. 3d) confirmed that under the “weight” condition, the mice could rarely walk and spent more time resting due to the burden of the additional weight.

Furthermore, we investigated the neural activities of the three mice based on their neuronal firing. Figure 3f shows the segmentation mask of 234 neurons identified in the field of view (FOV) of Mouse 1. Figure 3g plots the representative ΔF/F traces of 10 neurons among these identified neurons, with colored shading marking the duration of walking. We defined the fluorescence rate of a neuron as the area under its ΔF/F trace during walking divided by the total walking duration. Since the mice barely walk under the “weight” condition, we calculated the average fluorescence rate of all neurons during the walking duration for the “probe-only” and “balloon + weight” conditions. The results (Fig. 3e) indicated that the neuronal firing rates during walking were similar in the “balloon + weight” and “probe-only” conditions. This suggests that the added weight did not affect general neural activities, as the balloon’s buoyancy effectively alleviated the head-mounted weight burden.

## 4. Discussion

We established a buoyancy levitation system to alleviate head-mounted weight burden for neural imaging in freely-behaving mice. The experimental results demonstrated that the buoyancy-based method can effectively counteract a weight of up to 7 g when using a regular-size (15L) balloon inflated with a helium mixture. Employing a larger balloon could cancel out even heavier weights.

The performance of the system was evaluated by conducting neuron imaging in freely-behaving mice under three conditions: (1) Probe-only, (2) Balloon + Weight and (3) Weight. Analysis of the mice’s motion behavior revealed that the animals had similar freedom of movement when using a balloon to lift the head-mount 6 g weight compared to when only a fiberscope was mounted on their heads. In contrast, the mice could barely walk and needed more rest without a balloon to alleviate the weight burden. Additionally, by calculating the average fluorescence rate of neurons (somatosensory cortex) during the walking duration, we observed that the neurons exhibited a similar level of firing when the weight was canceled out by a balloon or only a probe was mounted. These results indicate that the buoyancy levitation system effectively alleviates the head-mounted weight burden on the mice. The buoyant force cancels the added weight, resulting in no perceptible difference in general neuronal firing activities.

Further investigation can explore the influence of weight burden on specific brain regions and enhance our understanding of the connection between head or neck muscles and corresponding neurons. We anticipate that our system will serve as a valuable tool for alleviating the weight burden on the animal’s head, offering new opportunities for studying neural circuit function under naturalistic conditions.

## Data Availability

The datasets used and/or analyzed during the current study are available from the corresponding author on reasonable request.
